# A multi-ethnic polygenic risk score for chronic kidney disease is associated with increased risk of hypertension in African American individuals

**DOI:** 10.21203/rs.3.rs-6674221/v1

**Published:** 2025-06-24

**Authors:** Aastha Kakar, Elizabeth M. Litkowski, Ashley W. Scadden, Mohammad Y. Anwar, Iain R. Konigsberg, Maggie A. Stanislawski, Natalie C. DuPre, Riten Mitra, Richard Baumgartner, Laura M. Rsffield, Ethan M. Lange, Leslie A. Lange, Kira C. Taylor

**Affiliations:** University of Louisville; University of Colorado Anschutz Medical Campus; University of Colorado Anschutz Medical Campus; University of North Carolina at Chapel Hill; University of Colorado Anschutz Medical Campus; University of Colorado Anschutz Medical Campus; University of Louisville; University of Louisville; University of Louisville; University of North Carolina at Chapel Hill; University of Colorado Anschutz Medical Campus; University of Colorado Anschutz Medical Campus; University of Louisville

**Keywords:** Polygenic risk scores, Chronic kidney disease, Hypertension, African American individuals

## Abstract

**Background:**

Hypertension (HT) and chronic kidney diseases (CKD) are complex conditions having both genetic and environmental contributions, disproportionately affecting African American (AA) individuals. Recent evidence is contradictory regarding the directionality of the relationship between the two conditions. This study investigates the relationship between CKD and blood pressure (BP)-related traits with CKD and BP by generating polygenic risk scores (PRSs) for CKD and BP-related traits in 2,995 AA participants of the Jackson Heart Study.

**Methods:**

We used multivariable regression models to evaluate associations of each PRS with CKD, HT, systolic blood pressure (SBP) and diastolic blood pressure (DBP), adjusting for age, sex, and genetic ancestry.

**Results:**

We observed positive associations for the CKD PRS (CKD-PRS) with both CKD (OR per standard deviation increase, 95% CI: 1.85, 1.64–2.09) and HT (1.10, 1.01–1.20). Adding the CKD-PRS to a multivariable model for CKD increased the area under the receiver operating curve (ROC) curve by 0.061. The CKD-PRS was also positively associated with DBP (beta = 0.37 mmHg, 95% CI: 0.01–0.73). The BP-PRSs were positively associated with HT, SBP and DBP; however, they were not associated with CKD.

**Conclusions:**

Our results indicate that genetic predisposition to CKD may increase the risk of hypertension in AA individuals. Our results also align with previous studies in European ancestry individuals that fail to support the causative role of blood pressure in kidney function decline, as we did not find an association between the blood pressure risk scores with CKD. Finally, we found a strong association between the CKD risk score with CKD in AA individuals, supporting its clinical use in an AA population. Overall, our findings provide valuable insights into the genetic underpinnings of CKD and HT in AA individuals.

## BACKGROUND

Chronic kidney disease (CKD) is a complex disease associated with higher risk of cardiovascular disease, end stage kidney disease (ESKD), disability, and all-cause and premature mortality. CKD is defined as a progressive loss of renal function and is associated with irreversible pathological changes in the kidney. The prevalence of CKD is disproportionately high among African American (AA) individuals – 16.3% as compared to 12.7% for non-Hispanic White individuals in the US.(1) The global prevalence of CKD in 2017 was 9.1% (697.5 million cases) and is estimated to increase to 16.7% by 2030 due to a rise in leading risk factors, such as hypertension and diabetes(2),(3). CKD is characterized as having both genetic and environmental contributions and has a strong heritable genetic component within the range of 25–75%(4, 5)

Hypertension (HT) is another complex disease associated with multiple interacting contributors such as lifestyle, environmental and genetic factors, and is the leading cause of worldwide coronary heart disease eventsand premature deaths, and is also associated with kidney disease.(6) It is also a heritable trait, ranging from 30–70%,(7) influenced by multiple biological pathways. Moreover, there is a disparity in the distribution of HT in different populations, with AA disproportionately affected.(8) The prevalence of hypertension in non-Hispanic Black adults was 56% and in non-Hispanic White adults was 48% in 2017.(9)

Previous studies have attributed the higher prevalence of CKD and HT in AA to the higher prevalence of risk factors such as obesity, smoking, alcohol consumption, social factors, and genetic predisposition.(10, 11) Many genetic variants have been found to be associated with increased risk of CKD and HT in recent epidemiological studies through various approaches, e.g., and comparative genomics. More than 100 loci associated with blood pressure (BP) and around 100 genetic loci associated with kidney function have been identified from GWAS, including genetic variants in or near *APOL1, UMOD, SHROOM3* and E3 ubiquitin ligases.(12-14) The majority of these GWAS have been conducted in primarily European ancestry populations, though some of the risk variants have been identified specifically in AA, such as *ARMC5* for HTN and *APOL1* for CKD.(15) However, the individual genetic variants display small effect sizes and only partially explain the heritability of these traits.

Polygenic risk scores (PRS) represent an approach that allows testing of a cumulative score that incorporates all the genetic variants that have been previously identified to be associated with the trait. As the cumulative burden of modest effect variants can result in substantial increased risk for those with scores in the upper tails of PRS distributions, PRS can be useful in identifying a subset of individuals with high risk of developing HT and CKD.

Kidney function and BP are known to be interrelated and previous literature suggests that they both may be a cause and a consequence of each other, implying a bidirectional relationship.(14, 16) However, recent genetic studies suggest that HT may be a consequence of sub-clinical kidney disease rather than a cause.(17, 18) A Mendelian randomization study conducted by Yu et.al. in European-ancestry individuals demonstrated that lower kidney function is causal to HT but high BP was not causally associated with kidney function.(19) In a study by Nierenberg et al., a BP-PRS was investigated with respect to CKD progression in the Chronic Renal Insufficiency Cohort (CRIC) study participants consisting of both African- and European-ancestry participants, which also demonstrated that the genetic contribution to BP was not associated with CKD progression in CKD patients.(20)

The present study aimed to achieve additional evidence regarding the relationship between CKD and HT by generating PRSs for CKD and BP-related traits, to evaluate their associations with both CKD as well as BP-related traits in an exclusively AA population, the Jackson Heart Study. To our knowledge, there have not been any similar studies published using an AA population.

## METHODS

### Study population

The study population was selected from the Jackson Heart Study (JHS), a community-based longitudinal cohort study consisting of 5,306 AA individuals from Jackson, Mississippi.(21) The age range at enrollment was 35–84 years old, except in a nested family cohort where the age range was extended to 21–84 years. Extensive medical and social history, phenotypic data as well as blood samples for genomic data were collected during the baseline examination (September 2000 –March 2004), and two follow-up examinations (October 2005–December 2008, and February 2009–January 2013). The JHS design, methods,(21) the study design for genetic analysis,(22) physical activity assessment methods(23) as well as sociocultural methods(24) have been published previously (Supplementary methods, Additional File 1).

For the present study, phenotypic observations from visit 1 were used (N = 5,306). After excluding participants with incomplete genetic, HT and CKD data, the total sample size used for this study was N = 2,995 individuals (*Supplementary Figure S1, Additional File 1*).

### Genotyping

JHS genome-wide genetic data (Affymetrix 6.0 SNP Array; Affymetrix, Santa Clara, CA) imputed to the 1000 Genomes Phase 3 (version 5) reference panel was used for this study.(25) Variant inclusion criteria were: minor allele frequency ≥ 1%, call rate ≥ 90%, and Hardy Weinberg equilibrium (HWE) p-value > 10^−6^ (n = 832,508 variants). Variants with invalid or mismatched alleles for the reference panel were removed prior to imputation. Imputation was completed using Minimac3 on the Michigan Imputation Server.

### Polygenic risk scores

Our analysis utilizes previously published polygenic risk scores which were developed and validated in an AA population for SBP and DBP and a multi-ethnic population for CKD (Supplementary methods, Additional File 1).(26),(27) These three PRS were applied to the JHS participants.

Three PRS were constructed to test their association with CKD and BP related traits: (1) PRS for CKD (CKD-PRS) (2) PRS for SBP (SBP-PRS) and (3) PRS for DBP (DBP-PRS). A weighted PRS was calculated as the sum of risk alleles at each locus multiplied by their corresponding genotype effect size estimates, where the effect sizes were obtained from the previously published GWAS summary statistic data.(26),(28)

PRS for CKD, SBP and DBP were calculated for each JHS participant using the PRSice-2 program, a computational tool to facilitate the calculation of PRS.(29) (without p-thresholding and clumping to allow for the inclusion of all available SNPs from the published risk scores described above). Standard quality control steps were conducted for both the GWAS summary statistics, as well as the JHS imputed genetic data, ensuring the same genome build assignment and effect allele designation. SNPs with low imputation information score (< 0.5) were excluded, as well as duplicates, indels, multiallelic SNPs, ambiguous SNPs, and mismatched SNPs. SNPs with low MAF were not removed to maximize the similarity between the risk scores used in this study with the originally published risk scores, to enable direct comparison of results. After applying these criteria, the total number of SNPs in the CKD, SBP and DBP risk scores were 470,378, 104,821 and 159,234 respectively. The final scores were standardized by subtracting the mean of the PRS and dividing by the standard deviation of each score.

### Outcomes

The dependent variables for the study were: CKD status (yes/no), HT status (yes/no), SBP (mmHg), and DBP (mmHg).

CKD was defined according to the National Kidney Foundation (NKF) guidelines as estimated glomerular filtration rate (eGFR) less than 60 mL/min/1.73 m^2^ or the presence of albuminuria, or dialysis therapy(30) where the eGFR values were based on the CKD-Epi equation.(31) The presence of albuminuria was calculated based on urine albumin to urine creatinine ratio (ACR) using spot urine values (ACR > 30 mg/g).(32)

HT was defined as SBP ≥ 140 mm Hg, DBP ≥ 90 mm Hg, or being on antihypertensive medications regardless of BP measurements. Participants were categorized as taking antihypertensive medication if they self-reported HT medication use or reported taking any medication used for treating HT during the past two weeks. For individuals on antihypertensive medication (52% of the sample), 15mmHg and 10mmHg was added to SBP and DBP respectively in the analysis for the models which used SBP or DBP as the outcome.(33-36)

### Covariates

Covariates included were age, self-reported sex, and the first 10 genetic principal components (PCs) to control for population substructure.

### Statistical analysis

We assessed SBP and DBP for consistency with normality using standard histograms. We used Spearman correlation and t-tests to assess associations between the BP traits with continuous and dichotomous baseline characteristics, respectively. Baseline characteristics of participants with and without HT and CKD were compared using χ^2^ tests for categorical variables and analysis of variance (ANOVA) for continuous variables, respectively, using the R package tableone v. 0.13.0.

Multivariable linear regression models were used to test each PRS as predictors of SBP and DBP. Logistic regression was used to test the association of each PRS with CKD and HT. We calculated effect estimates for a 1 standard deviation (SD) unit change in PRS, and for deciles of risk scores. Area under the receiver operating curve (AUROC) was calculated for models with and without the polygenic risk scores for the dichotomous outcomes. For the continuous outcomes, we calculated the proportion of variance explained (multiple R^2^) for full models (PRS plus covariates) and for covariates-only models. Standardized CKD-, SBP- and DBP-PRSs were tested for all four outcomes. Considering that the GWAS studies did not have the same proportion of males and females as the JHS, we also investigated interaction of each PRS with sex by including product terms in the models. All models were adjusted for age, sex and the first ten genetic PCs.

To compare the AUROC, or discriminatory ability of the models with the PRSs vs. those without, the R package pROC v.1.18.0 was used to calculate the AUC for each model. AUC was calculated by subsetting 70% of the total sample as the training set (n = 2,100) and the remaining 30% of the sample as the testing set (n = 895), followed by fitting logistic regression models for models with PRS plus covariates (age, sex and 10 PCs) vs covariates-only model and lastly, calculating the probability of default for each individual in the testing dataset. Finally, we performed a series of analyses including combinations of the PRSs for the four outcomes to assess whether including multiple scores improved prediction of our outcome measures compared to analyses including a single PRS. All statistical analyses were performed using R (version 4.0.2, R Foundation for Statistical Computing, Vienna, Austria). A p-value of < 0.05 was considered statistically significant.

## RESULTS

### Population characteristics

Participants had a mean age of 54 years and 38% were male (Table 1). Approximately 12% of the participants had CKD, and 60% of the participants had HT. Among the 2,995 participants included in the study, participants with CKD were more likely to be older, female, obese, non-smoker, and to have type 2 diabetes (55%), compared to those without CKD. Individuals with and without HT had a similar pattern to CKD with respect to these baseline characteristics (*Supplementary Table S1, Additional File 1*). The distributions for the CKD, SBP and DBP PRS in JHS are presented in *Supplementary Figure S2, Additional File 1*.

### Association Results

#### Association of PRS with their respective outcomes

##### CKD-PRS

**Figure 1:**
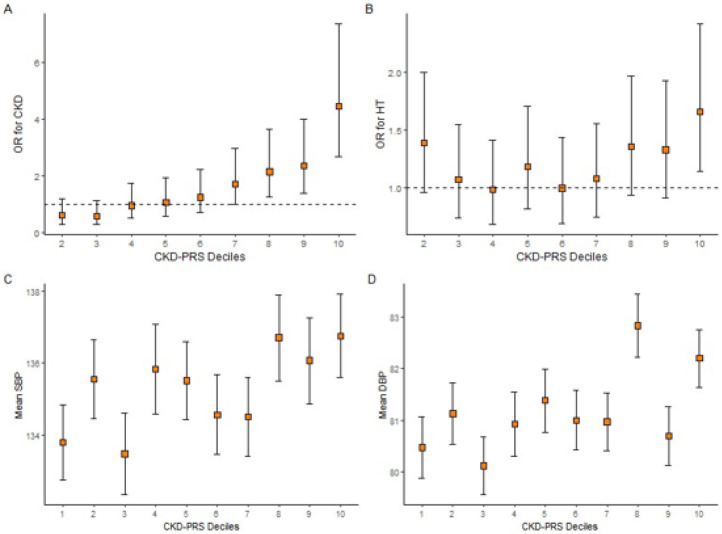
CKD-PRS Deciles. Top (A,B): OR for chronic kidney disease (CKD) and hypertension (HT) status by deciles of CKD-PRS in JHS. Error bars indicate confidence intervals of the odds ratio; reference decile was set to decile 1. Bottom (C,D): Relationship between mean SBP and mean DBP with deciles of CKD-PRS in JHS. Error bars indicate standard error of the mean.

The CKD-PRS was positively associated with CKD. For a one SD unit increase in the CKD-PRS, the odds of CKD increased by 85% (95% confidence interval [CI]: 1.64–2.09). Furthermore, the association of the CKD-PRS deciles with CKD showed a strong positive trend. Individuals in the highest decile have over four times the odds of having CKD versus individuals in the lowest decile (95% CI: 2.73–7.45; p < 0.001) (Fig. 1 and *Supplementary Table S2, Additional File 1*).

Performance of the models including the CKD-PRS and primary covariates (age, sex and 10 genetic PCs) revealed an area under the receiver-operator curve (AUC) of 0.728 (95% CI: 0.668–0.788), an increase of 0.061 from the covariate-only model (AUC (95% CI) = 0.667 (0.606–0.729)) for the CKD outcome (*Figure S3*).

##### SBP-PRS

**Figure 2:**
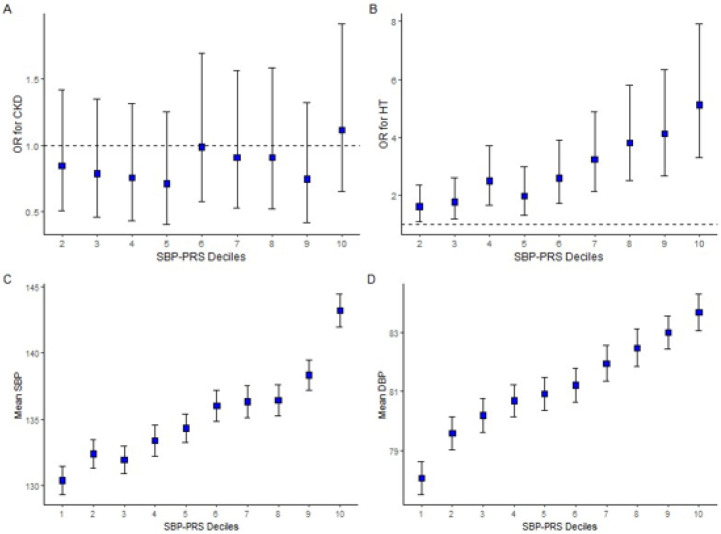
SBP-PRS deciles. Top (A,B): OR for chronic kidney disease (CKD) and hypertension (HT) status by deciles of SBP-PRS in JHS. Error bars indicate confidence intervals of the odds ratio; reference decile was set to decile 1. Bottom (C,D): Relationship between mean SBP and mean DBP with deciles of SBP-PRS in JHS. Error bars indicate standard error of the mean.

The SBP-PRS was positively associated with HT, SBP and DBP. For a one SD increase in SBP-PRS, the odds of HT increased by 59% (95% CI: 1.43–1.77) and the systolic and diastolic BPs increased by 4.51mmHg (95% CI: 3.77–5.25) and 1.89 mmHg (95% CI: 1.46–2.31), respectively (Table 2). Additionally, a positive trend was observed between higher SBP-PRS deciles and the odds of HT as well as with mean SBP and DBP; the individuals in the highest SBP-PRS decile had 5-fold increased odds for having HT as compared to the individuals in the lowest decile (95% CI: 3.32–7.93) and had an estimated increase of 16.51 mmHg (95% CI: 13.43–19.60) and 6.66 mmHg (95% CI: 4.89–8.43) in SBP and DBP, respectively (Fig. 2; *Supplementary Tables S6-9, Additional File 1*).

When the models were compared with and without the SBP-PRS, the AUC for HT marginally increased with the inclusion of SBP-PRS: AUC = 0.775 (95% CI: 0.743–0.806) compared with 0.764 (95% CI: 0.732–0.796), an increase of 0.011. (*Figure S4*). The SBP-PRS component alone explained 3.59 and 2.41 percent of the variance of SBP and DBP, respectively (*Supplementary Tables S16,17, Additional File 1*).

##### DBP-PRS

**Figure 3:**
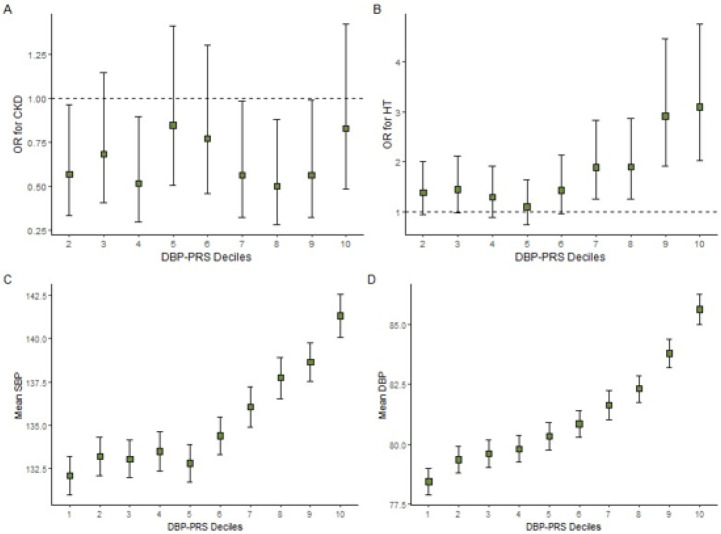
DBP-PRS deciles. Top (A,B): OR for chronic kidney disease (CKD) and hypertension (HT) status by deciles of DBP-PRS in JHS. Error bars indicate confidence intervals of the odds ratio; reference decile was set to decile 1. Bottom (C,D): Relationship between mean SBP and mean DBP with deciles of DBP-PRS in JHS. Error bars indicate standard error of the mean.

The regression results of association of DBP-PRS with HT, SBP and DBP were similar to those for SBP-PRS. The DBP-PRS was positively associated with HT, systolic- and diastolic BPs. For a one SD increase in DBP-PRS, the odds of HT increased by 40% (95% CI: 1.26–1.55) and the systolic and diastolic BPs increased by 3.02 mmHg (95% CI: 2.25–3.80) and 2.53 mmHg (95% CI: 2.10–2.96; p < 0.001) respectively (Table 2). The DBP-PRS deciles showed significantly higher odds of HT and greater means of SBP and DBP in the upper deciles relative to the lowest decile group; the individuals in the highest DBP-PRS decile had a three-fold increased odds of having HT compared to the individuals in the lowest decile (95% CI: 2.02–4.76; p < 0.001) and had an increase of 10.96 mmHg (95% CI: 7.74–14.06; p < 0.001) and 8.43 mmHg (95% CI: 6.65–10.20; p < 0.001) for SBP and DBP, respectively (Fig. 3).

The AUC for the model with the DBP-PRS was 0.768 (95% CI: 0.7366–0.7999) compared with the AUC 0.764 (95% CI: 0.732–0.796) for the covariates-only model, a minimal increase of 0.004 (*Figure S4*). The DBP-PRS component alone explained 1.51 and 4.06 percent of the variance of SBP and DBP, respectively (*Supplementary Tables S16,17, Additional File 1*).

#### Association of CKD-PRS with BP-related traits

The odds of HT increased by 10% (95% CI: 1.01–1.20); and mean DBP increased by 0.37 mmHg (95% CI: 0.01–0.73) with a one SD increase in the CKD-PRS (Table 2). The association of CKD-PRS with SBP had a larger effect size than with DBP, but a larger standard error (beta: 0.55 mmHg; 95% CI: −0.09-1.19). A clear trend was not observed when examining the CKD-PRS deciles with the blood pressure traits (Fig. 1; *Supplementary Tables S3-5, Additional File 1*). The CKD-PRS component of the multivariable model explained 0.07 and 0.13 percent of the variance of SBP and DBP, respectively (*Supplementary Tables S16,17, Additional File 1*).

#### Association of the BP-PRS with CKD

The SBP-PRS was not associated with the odds of CKD (OR: 1.05, 95% CI: 0.91–1.20), and the decile analysis was consistent with this observation (Fig. 2; *Supplementary Tables S6-9, Additional File 1*). The DBP-PRS was not associated with CKD (0.97, 95% CI: 0.84, 1.12) (Table 2). Additionally, some deciles of DBP-PRS were inversely associated with CKD; however, there was no clear pattern, and a linear trend was not observed (*Supplementary Tables S10-13, Additional File 1*).

### Combining multiple PRS

When combining multiple PRS in the prediction models, we observed no significant improvement in our prediction models compared to models that included only the corresponding single PRS (e.g. the prediction of CKD using CKD-PRS alone was not improved when including SBP-PRS and DBP-PRS to the model) (*Supplementary Table S14, Additional File 1*). DBP-PRS and SBP-PRS were strongly positively correlated with each other and were both weakly positively correlated with the CKD-PRS (*Supplementary Figure S5, Additional File 1*). Of note, for HT, while both SBP-PRS and DPB-PRS were strongly associated with HT when analyzed individually, including both SBP-PRS and DBP-PRS in the prediction model resulted in the effect of SBP-PRS remained strong while the effect of DBP-PRS was attenuated (*Supplementary Table S15, Additional File 1*).

### Sex interaction

We did not observe significant interaction between the three PRS and sex on any of the outcomes (P_interaction_>0.05 for all tested interaction terms).

## DISCUSSION

In the current study, we investigated the relationships of three PRS (CKD, SBP, and DBP) with development of CKD and BP-related traits in AA. As expected, we observed a strong positive association between the CKD-PRS and CKD. We also observed positive associations for the BP-PRSs with HT, SBP and DBP, as expected. The CKD-PRS was also positively associated with HT and DBP. However, there was no association between either of the BP risk scores and CKD. Our results are consistent with recently published studies in European ancestry individuals that fail to support the causative role of BP in kidney function decline.(18-20)

When the associations between the CKD-PRS and BP traits were tested, we observed small effects of CKD-PRS on DBP and HT, but not on SBP. The decile analysis suggests these associations were driven by those in the highest deciles (highest genetic risk). These results imply that individuals with a strong genetic predisposition to CKD are at increased risk of having HT. While these results may also be partially due to the potential overlap of SNPs in the CKD and BP risk scores, CKD-PRS and BP-PRS were only weakly positively correlated (Pearson correlation coefficient = 0.1, *Supplementary Figure S5, Additional File 1*), and the converse effect was not observed (i.e., there was no association between the BP-PRSs and CKD). We did not find other studies that tested associations between CKD-PRS and BP outcomes in AAs.

We did not observe any evidence for associations between either BP-PRS with CKD. Our results are consistent with PRS studies by Ehret et.al and Nierenberg and colleagues in European and African ancestries,(18, 20) as well as the Mendelian randomization study by Yu et. al that also reported absence of association between the BP risk score and CKD.(19) However, our results are in contrast with a Mendelian randomization study by Staplin et al. in European participants which found a significant association between a SBP-PRS and glomerular hyperfiltration, a precursor to CKD.(37) The main difference between the Staplin study as compared to other previous Mendelian randomization (MR) studies was the use of nonlinear MR models by Staplin et al., displaying a nonlinear association between BP and eGFR.

There are different hypotheses that have been suggested for explaining discrepancies in results from studies assessing BP and CKD directionality. Some have hypothesized that only severe HT would lead to kidney function decline since the kidney has the ability to adapt to small differences in BP.(38, 39) Others have hypothesized that the glomerular biotrauma may be due to peak glomerular perfusion pressure instead of mean perfusion pressure.(37) Our results are consistent with other epidemiological studies which also found that BP-PRSs were not associated with CKD.(20, 40) Additionally, results from recent Mendelian randomization studies also support the causal effects of higher kidney function on lower BP, but the causal effects of BP on kidney function have not been supported.(19, 41) These discrepancies leave the role of BP in the onset and evolution of kidney disease unresolved and additional studies are needed to further explore the association between the two.

### Strengths and Limitations

Our study exhibits several notable strengths. Firstly, our study was based on data from participants from the JHS, a prospective cohort study that adhered to standardized data collection protocols for both phenotype and genetic data. Our study's rigor also extends to the outcome definitions, which were harmonized with those used in the previous GWAS used to construct the PRS, ensuring consistency and reliability in our assessment of outcomes. An additional advantage of using JHS is its exclusive inclusion of AA participants, addressing the underrepresentation of this population in genomics research. AA are at higher risk for both HT and CKD, increasing the potential for a meaningful public health impact of this research. Additionally, the SBP- and DBP-PRS were derived from the summary statistics of a large, AA GWAS. Using a PRS generated from a large AA GWAS and applying it to AA participants in JHS should increase the validity of the results. Moreover, normal-standardized scores were utilized for the analysis, offering several advantages, including improved comparability, interpretability, and utility in both research and clinical settings.

However, this study also has several limitations that should be noted. Regarding the studies used to generate the PRS, the distribution of sex in the discovery studies is different than in the JHS – the MVP study has 91% males, whereas JHS has 36.5% males for Visit 1. If the associated BP genes have a stronger association in males, then this may result in attenuated effects in our study. However, we examined whether sex modified PRS have effects on outcomes and did not observe significant interactions between the two predictors. Additionally, COGENT includes JHS participants which could lead to overestimation of the effect estimates. However, JHS represents only a very small proportion (2.1%) of the original GWAS sample. Regarding the CKD-PRS, the risk score was generated from a multiethnic GWAS where AA represented approximately 2% of the total sample. Since it is known that some of the genetic variants which are strongly associated with CKD (e.g., *APOL1*) are found in much higher proportions in AA, the effect estimates for the CKD-PRS may be underestimated. Regarding the phenotypes, analyses were based on single measurements of eGFR and albuminuria. Misclassification is thus a possibility for the CKD outcome, the main exposure variable (genotypes), and covariates. For example, assays for biomarkers such as creatinine may differ slightly across laboratories, which would impact classification of CKD status in participants. However, stringent QC criteria have been applied in each study, including JHS.

### Conclusion

In summary, this study of PRS in AA provides further support that BP elevation may be a consequence of having a genetic predisposition to CKD. Additionally, our results add evidence towards utilizing the CKD-PRS in clinical settings to identify AA individuals at high risk for CKD.

## Supplementary Material

This is a list of supplementary files associated with this preprint. Click to download.

AdditionalFile1.docx

## Figures and Tables

**Table 1:** Baseline characteristics of the JHS study participants Overall and by CKD status.

	All participants (N = 2,995)	CKD status		
		Without CKD (N = 2,637)	With CKD (N = 358)	p-value
**Age (years),** Mean (SD)	54.57 (12.87)	53.60 (12.54)	61.75 (13.00)	< 0.001
**Male**, n (%)	1145 (38.2)	1018 (38.6)	127 (35.5)	0.278
**Income**, Mean (SD)	2.79 (1.03)	2.82 (1.03)	2.55 (1.00)	< 0.001
**Current smoker**				0.029
No, n (%)	2563 (85.6)	2240 (84.9)	323 (90.2)	
Yes, n (%)	407 (13.6)	374 (14.2)	33 (9.2)	
Missing, n (%)	25 (0.8)	23 (0.9)	2 (0.6)	
**Alcohol consumption**				< 0.001
No, n (%)	1561 (52.1)	1327 (50.3)	234 (65.4)	
Yes, n (%)	1418 (47.3)	1294 (49.1)	124 (34.6)	
Missing, n (%)	16 (0.5)	16 (0.6)	0 (0.0)	
**Active Index,** Mean (SD)	2.07 (0.80)	2.09 (0.80)	1.90 (0.77)	< 0.001
**BMI (kg/m2),** Mean (SD)	32.01 (7.45)	31.87 (7.35)	33.02 (8.11)	0.007
**BMI Categories**				0.330
Normal Weight, n (%)	393 (13.1)	352 (13.3)	41 (11.5)	
Obese, n (%)	1624 (54.2)	1414 (53.6)	210 (58.7)	
Overweight, n (%)	963 (32.2)	859 (32.6)	104 (29.1)	
Underweight, n (%)	11 (0.4)	9 (0.3)	2 (0.6)	
Missing, n (%)	4 (0.1)	3 (0.1)	1 (0.3)	
**Diabetes**				< 0.001
No, n (%)	2279 (76.1)	2083 (79.0)	196 (54.7)	
Yes, n (%)	714 (23.8)	553 (21.0)	161 (45.0)	
Missing, n (%)	2 (0.1)	1 (0.0)	1 (0.3)	
**ACR,** Mean (SD)	52.29 (280.08)	7.34 (5.60)	281.16 (645.28)	< 0.001
**Albuminuria**				< 0.001
No, n (%)	1265 (42.2)	1227 (46.5)	38 (10.6)	
Yes, n (%)	203 (6.8)	0 (0.0)	203 (56.7)	
Missing, n (%)	1527 (51.0)	1410 (53.5)	117 (32.7)	
**eGFR**, Mean (SD)	94.51 (22.43)	98.01 (18.07)	68.72 (32.41)	< 0.001
**Dialysis Ever**				< 0.001
No, n (%)	2950 (98.5)	2608 (98.9)	342 (95.5)	
Yes, n (%)	14 (0.5)	0 (0.0)	14 (3.9)	
Missing, n (%)	31 (1.0)	29 (1.1)	2 (0.6)	
**BP meds**				< 0.001
No, n (%)	1394 (46.5)	1332 (50.5)	62 (17.3)	
Yes, n (%)	1577 (52.7)	1283 (48.7)	294 (82.1)	
Missing, n (%)	24 (0.8)	22 (0.8)	2 (0.6)	
**BP meds, self-reported**				< 0.001
No, n (%)	1456 (48.6)	1386 (52.6)	70 (19.6)	
Yes, n (%)	1482 (49.5)	1203 (45.6)	279 (77.9)	
Missing, n (%)	57 (1.9)	48 (1.8)	9 (2.5)	
**Hypertension (yes),** n (%)	1789 (59.7)	1478 (56.0)	311 (86.9)	< 0.001
**SBP**, Mean (SD)	127.37 (16.65)	126.43 (16.17)	134.33 (18.39)	< 0.001
**DBP**, Mean (SD)	75.91 (8.81)	75.93 (8.72)	75.72 (9.48)	0.668
**CKD (yes),** n (%)	358 (12.0)			

**Table 2:** Regression results for the effects of SBP, DBP, and CKD polygenic risk scores on blood pressure-related outcomes and CKD.

	Outcomes				
Exposures	SBP^a^	DBP^a^	HT^b^	CKD^b^
SBP-PRS	4.51 (3.77, 5.25)	1.89 (1.46, 2.31)	1.59 (1.43, 1.77)	1.05 (0.91, 1.20)
DBP-PRS	3.02 (2.25, 3.80)	2.53 (2.10, 2.96)	1.40 (1.26, 1.55)	0.97 (0.84, 1.12)
CKD-PRS	0.55 (−0.09, 1.19)	0.37 (0.01, 0.73)	1.10 (1.01, 1.20)	1.85 (1.64, 2.09)

a Effects shown are beta estimates and 95% CI from linear regression, for a one SD increase in the PRS.

b Effects shown for dichotomous outcomes are odds ratios and 95% CI, for a one SD increase in the PRS.

Model adjusted for age, sex and first 10 ancestry principal components.

**PRS**: Polygenic risk score, **SBP**: Systolic blood pressure, **DBP**: Diastolic blood pressure, **CKD**: Chronic kidney disease, **HT**: Hypertension, **β**: effect size (change in SBP or DBP (mmHg) per one standard deviation increase in risk score for CKD)

## Abbreviations

AA: African American
ACR: Urine albumin to urine creatinine ratio
AUC: Area under the curve
AUROC: Area under the receiver operating curve
BMI: Body mass index
CKD: Chronic kidney disease
CKD-PRS: Polygenic risk score for chronic kidney disease
COGENT: Continental Origins and Genetic Epidemiology Network
CRIC: Chronic Renal Insufficiency Cohort
DBP: Diastolic blood pressure
DBP-PRS: Polygenic risk score for diastolic blood pressure
eGFR: estimated glomerular filtration rate
ESKD: End-stage kidney disease
GWAS: Genome-wide association studies
HT: Hypertension
HWE: Hardy Weinberg equilibrium
JHS: Jackson Heart Study
MAF: Minor allele frequency
MVP: Million Veterans Program
MR: Mendelian randomization
OR: Odds ratio
PCs: Principal components
PRS: Polygenic risk score
ROC: Receiver operating curve
SBP: Systolic blood pressure
SBP-PRS: Polygenic risk score for systolic blood pressure
SNPs: Single-nucleotide polymorphisms
